# Pre-morbid statin use and mortality in trauma: a systematic review and meta-analysis

**DOI:** 10.1007/s00423-026-04011-8

**Published:** 2026-04-02

**Authors:** Andrew Markle, Ravi Vissapragada, Kaethe Sabr, Zane B. Perkins, Henry D. De’Ath

**Affiliations:** 1 Liver Intensive Therapy Unit, King’s College Hospital, London, UK; 2 Department of Upper Gastrointestinal Surgery, Frimley Park Hospital, Camberley, UK; 3 Humanitarian and Conflict Response Institute, University of Manchester, Manchester, UK; 4 Centre for Trauma Sciences, Queen Mary University of London, London, UK; 5 Flinders University, Adelaide, Australia

**Keywords:** HMG-CoA reductase inhibitor, Injury, Traumatic brain injury, Burns, Secondary injury

## Abstract

**Purpose:**

Secondary injury after trauma is responsible for significant morbidity and mortality. Inflammation appears to play a central role. Some evidence proposes that statins (HMG-CoA reductase inhibitors) may modulate this inflammation via their pleiotropic properties (non-cholesterol lowering effects). These include suppression of complement activation, vasodilation and inhibition of platelet function and aggregation. The purpose of this systematic review and meta-analysis was to investigate whether pre-morbid statin use is associated with differences in outcomes after trauma.

**Methods:**

MEDLINE, EMBASE, Central, Google Scholar, clinicaltrials.gov, clinicaltrialsregister.eu and the German clinical trials register were searched for articles published between 01.09.1987 and 31.12.2023 examining pre-morbid statin use on outcomes after trauma.

**Results:**

After removal of duplicates, 623 records for abstract review were identified, of which nine were included in the systematic review and eight the meta-analysis. All studies were retrospective and most did not confirm in-hospital administration of pre-morbid statins. All had a high risk of bias. Grading of Recommendations Assessment indicated a very low certainty of results. When considering pre-morbid statin use in all types of trauma, the overall mortality risk ratio was 0.66 (95% CI 0.37–1.17) with high heterogeneity (I2 = 99%). The use of statins before traumatic brain injury (TBI) indicated a mortality risk ratio of 0.60 (95% CI 0.28–1.27, I2 = 96%). Analysis non-TBI studies yielded a risk ratio of 0.84 [95% CI 0.56–1.27].

**Conclusion:**

In a limited meta-analysis, data failed to demonstrate an association between pre-morbid statin use following serious injury. As this may be due to a heterogeneity in data, variable study population, and a number of confounding factors, further dedicated study is warranted to clarify whether observed associations reflect pharmacologic effects.

**Supplementary Information:**

The online version contains supplementary material available at 10.1007/s00423-026-04011-8.

## Introduction

Statins are one of the most commonly prescribed chronic medications [1, 2]⁠, with both cholesterol lowering and pleiotropic actions. Pleiotropic effects may moderate secondary insult after injury, however, this effect has not been well studied. The World Health Organisation (WHO) estimates that approximately 9% of worldwide deaths occur as a result of severe trauma [3]⁠. Injury remains one of the ten commonest causes of death in low-income to upper-middle income countries [4]⁠. Although the only effective strategy for reducing the impact of primary injury is education and prevention, complications and secondary injuries are inviting therapeutic targets for health systems. A 2017 Lancet study estimated the global burden of injury in disability-adjusted life years (DALY) is over 250 million [5]⁠. Further, that patients with moderate to severe disability survive longer in areas with developed health care systems.

Lovastatin was the first statin developed and was initially marketed in 1987 [6]⁠. Statins (HMG-CoA reductase inhibitors) are now among the most commonly prescribed medications in western Europe [1, 2]⁠⁠. Statins reversibly inhibit the formation of mevalonic acid and subsequently cholesterol, leading to “Reductions in all-cause mortality, major vascular events and revascularisations with no excess of adverse events among people without evidence of cardiovascular disease treated with statins” [7]⁠.

Statins also demonstrate pleiotropic properties, namely non-cholesterol-lowering effects of HMG-CoA reductase inhibitors (Appendix S1). Beyond trauma, statins have been investigated in the context of sepsis and septic shock, where dysregulated inflammation, endothelial dysfunction, and microvascular thrombosis are central to pathophysiology. Observational studies and early interventional trials have suggested that pre-morbid statin use may be associated with reduced mortality and organ dysfunction in sepsis and septic shock, potentially mediated through immunomodulatory, endothelial, and antithrombotic effects [8]. Although randomised trials have yielded mixed results, these findings provide biological plausibility for a potential association between statin exposure and outcomes following severe systemic insults, including trauma-related secondary injury.

Several have examined these in the context of moderating secondary insult after injury. A 2010 Danish study of over 12,000 intensive care unit (ICU) patients demonstrated that pre-morbid statin use correlated with both a significant reduction in all-cause 30-day [mortality rate ratio (MRR) 0.76 (95% CI 0.68–0.85)] and one-year mortality [MRR 0.70 (95% CI 0.63–0.80)] [9]⁠. Although theoretical benefits exist, whether pre-morbid statin exposure is associated with improved clinical outcomes after trauma remains uncertain, and causal inference is not possible from the observational evidence. The aim of this systematic review and meta-analysis was to examine any associations of pre-morbid statin use with improved survival and overall outcomes in patients after trauma specifically whether the estimated MRR for statin users is less than 1 compared with non-users.

## Materials and methods

The protocol for this meta-analysis was prospectively registered with PROSPERO (CRD42021228656) and reported according to PRISMA guidelines.

### Search strategy

Three investigators (AM, KS, RV) searched MEDLINE, EMBASE, Central, Google Scholar, clinicaltrials.gov, clinicaltrialsregister.eu and the German clinical trials register for articles published between 01.09.1987 and 31.12.2023 in English, French and German. Combinations of the following terms in both Medical Subject Heading (MeSH) and free text forms were used: statin or HMG-CoA reductase (inhibitor) and injury or (poly) (multi) trauma. Identified articles (including their reference lists) were manually searched to identify additional publications. If a register of trials identified a study without linking to the article, the author was contacted.

### Eligibility

Articles examining outcomes following trauma in humans with pre-morbid statin therapy were sought. Animal studies, studies without control groups, studies initiating statin therapy as an intervention and those involving pregnant patients were excluded.

### Study review and selection

Three reviewers (AM, KS, RV) examined article titles and abstracts and reviewed potentially eligible articles in full-text form. A fourth reviewer (HDD) was available to resolve disagreements.

### Data extraction

Data was compiled using the Systematic Review Data Repository [10].

### Quality of evidence and risk of bias assessment

Methodological quality of studies was appraised for quality using the Quality Assessment Tool for Observational Cohort and Cross-Sectional Studies [11]⁠. Risk of bias was assessed using the Cochrane Collaboration’s Tool to Assess Risk of Bias in Cohort Studies as well as visually using a funnel plot. Certainty of Evidence was done through Grading of Recommendations Assessment (GRADE). As all included studies were retrospective observational cohorts, the certainty of evidence for each outcome initially started at low. Evidence was subsequently downgraded across the following prespecified domains: risk of bias, inconsistency, indirectness, imprecision, and publication bias.

### Outcomes

Pre-morbid statin exposure was defined according to each included study’s operational criteria, which in most cases relied on prescription records, administrative coding, or pre-admission medication lists. Primary outcome was mortality. Secondary outcomes included ICU and hospital length-of-stay and infection/sepsis. Other commonly reported outcomes would be examined on a post-hoc basis where data was available.

### Statistical analysis

A limited meta-analysis was performed as a means to explore correlations instead of quantifying a pooled effect. Where only the relative risk was presented [12]⁠, the number of events were back calculated from the relative risk and sample size. Individual study weights were assigned using the Freeman-Tukey double arcsine transformation with inverse variance method. The statistical analysis was performed using R version 3.6.3 and Meta package (version 4.10, Schwarzer G.), employing the Mantel-Haenszel method and a random effects model due to the extreme heterogeneity of the included studies.

Using the random effects model, TBI and non-TBI subgroups were examined.

## Results

**Fig. 1:**
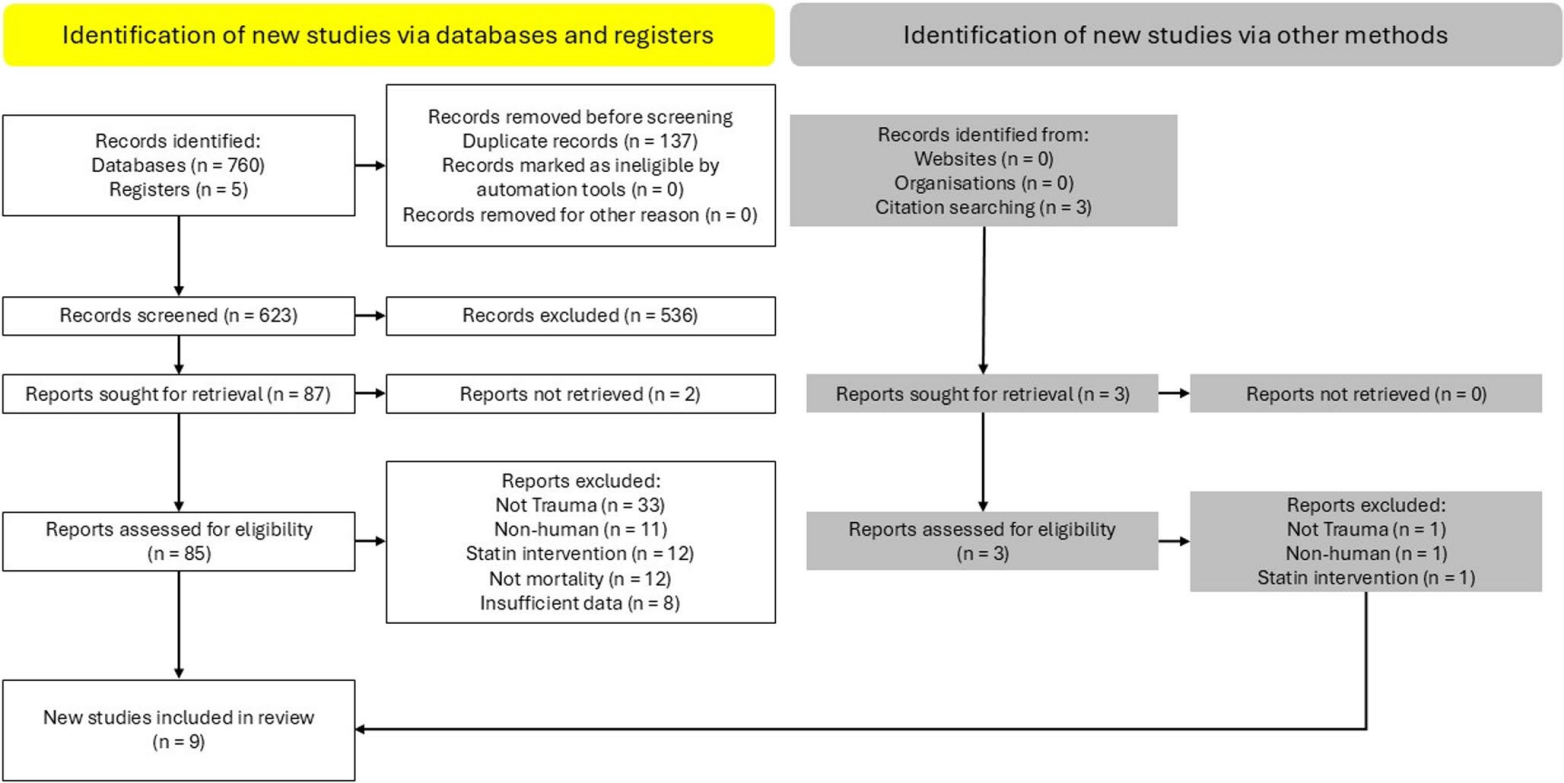
PRISMA flowchart

Nine studies were identified for inclusion in the systematic review, eight of which were included in the meta-analysis (PRISMA-Flowchart, Fig. 1). After removal of duplicates, we identified 628 records for abstract review. 541 were excluded. Of the eighty-seven reports eligible for full-text review, eighty-five were retrieved. The authors of the two remaining studies (one in report-form and the other in abstract) were contacted, but neither responded. One study was eliminated from the meta-analysis [13]⁠ as the data was reported using a statistical method that made accurate inclusion impossible. The author did not respond to enquiries. A manual search of relevant bibliographies identified three further articles for review. None were included in the final review or meta-analysis. A summary of the included studies can be found in Table 1. Due to significant heterogeneity, varying degree and mechanism of trauma, a limited meta-analysis was performed for hypothesis generation rather than assigning pooled effects of statin use in trauma patients and must be interpreted as such.

**Table 1:** Summary of included studies

Study	Design	Participants	Exposure	Comparison	Outcomes	Results	Statin continuation	Differentiates between statins	Comments
Fogerty et al 2010	Single center, retrospective cohort	Burn patients >55 years (n=223)	Pre-morbid statin use (n=70)	No pre-morbid statin use (n=153)	In-hospital mortality septic shock infection (not predefined)	OR 0.17 (95% CI 0.05-0.57) *p*0.004 OR 0.5 (95% CI 0.20-1.3) *p*=0.15 OR 0.9 (95% CI 0.48-1.7) *p*=0.75	77%	n	Multivariate analysis shows statins and septic shock are independent predictors of mortality.
Efron et al 2008	National database (NSCOT). retrospective cohort	Moderate to severe trauma (AIS ≥3) aged 65-84 years (n=1224)	Pre-morbid statin use (n=21.1 %) weighted n=529	No pre-morbid statin use (n=78.9% ) weighted n=1887	In-hospital mortality septic shock infection (not predefined)	OR 0.33 (95% CI 0.12-0.92) *p*0.04 With cardiovascular co-morbidity 1.4 (95% CI 0.72-2.72) *p*0.31 Without cardiovascular co-morbidity 0.3 (95% CI 0.1-0.91) *p*0.03 14.6% vs 20.7% *p*0.04	n	n	Complex statistical modelling effectively doubles the study population. Statin usage denned using only the class of medication. It is possible that some data points are duplicated in the Schneider et al study as the population is also derived from the NSCOT database.
Neal et al 2009	Multi-center. retrospective cohort	Patients >55 yrs suffering blunt trauma and BP<90mmHg or base deficit ≥6 mEq/L or blood transfusion within the 1^st^ 24 hrs. and AIS ≥2 excluding isolated head injury (n=295)	Pre-morbid statin use (n=71)	No pre-morbid statin use (n=224)	In-hospital mortality multi- organ failure infection	HR 1.98 (95% CI 0.9-4.0) *p*0.072 HR 1.81 (95% CI 1.1-2.9) *p*0.021 HR 0.78 (95% CI 0.5-1.4) *p*0.341	n	n	Data subset from the Host Response to Injury Large Scale Collaborative Program
Schneider et al 2011	Multi-center. retrospective cohort	TBI patients ≥65 yrs. With AIS ≥3	Pre-morbid statin use (n=242)	No pre-morbid statin use (n=723)	In-hospital mortality GOS-E good outcome @3 months @12 months	RR 0.24(95% CI 0.08-0.69) *p*? With cardiovascular co-morbidity RR 0.87 (95% CI 0.5-1.5) *p*? RR 0.77 (95% CI 0.42-1.41) *p*? RR 1.13 (95% CI 1.01-1.26) *p*?	n	n	Complex statistical modelling with weighting. It is possible that some data points are duplicated in the Efron et al study as the population is also derived from the NSCOT database.
Khokar et al 2017*	Retrospective cohort using insurance data	Medicare patients ≥65 yrs. Suffering TBI usingICD-9 codes(severity not defined) with atleast 6 months of Medicare coverage	Pre-morbid statin use (n=45752)	No pre-morbid statin use (n=66357)	In-hospital mortality 30-day mortality 60-day mortality	OR 0.87(95% CI 0.82-0.92) 30-day and 60-day mortality analyzed by statin (atorvastatin, rosuvastatin, atorvastatin)	n	y	Complex data harvesting involving different types of simultaneous Medicare coverage. Largest benefit seen with rosuvastatin.
Khokar et al 2018	Retrospective cohort using insurance data	Medicare patients ≥65 yrs. Suffering TBI usingICD-9 codes(severity not defined) with atleast 6 months of Medicare coverage	Pre-morbid statin use (n=50173)	No pre-morbid statin use (n=50342)	30-day mortality stroke depression ADRD	RR 0.32 (95% CI 0.31-0.33) RR 0.86 (95% CI 0.81-0.91) RR 0.85 (95% CI 0.79-0.90) RR 0.77 (95% CI 0.73-0.81)	n	y	Complex data harvesting involving diverse types of simultaneous Medicare coverage. Provides a partial 12-month analysis with similar results to the 30-day end point. Analyses the statins individually.
Neilson et al 2016	Single center, retrospective cohort	Patients suffering TBI (GCS <9)	Pre-morbid statin use (n=59)	No pre-morbid statin use (n=59)	14-day mortality GOS	OR 1.23 (95% CI 0.45-3.36) *p*68 OR 1.19 (95% CI 0.35-4.05) *p*78	n	n	A priori 6-month mortality outcome reported using only Kaplan-Meier curve.
Mcmahon et al 2018	Single center, retrospective cohort	Trauma patients admitted to a military trauma center and ISS >9 and ICU LOS >3 days	Pre-morbid statin use (n=565)	No pre-morbid statin use (n=1942)	In-hospital mortality VTE DVT (not predefined) ICU LOS days	43.2% vs 40.7% OR 1.82 (95% CI 1.2-2.8) p <0.01 OR 2.06 (95% CI 1.23-3.44) p <0.01 15.5 ±21.6(3-246) vs 7.9 ± 8.1(3-75) p<0.001	n	n	Data sampling relied on continuation of statins as an indicator of pre-morbid statin use. Statin users 12.8 yrs older than non-statin users.
Lokhandwala et al 2020	Single center, retrospective cohort	Isolated TBI patients ≥18 yrs. (extracranial AIS <3)	Pre-morbid statin use (n=90)	No pre-morbid statin use (n=180)	In-hospital mortality GOS-E score (IQR) ICU LOS days (IQR)	Overall, 21% vs 11% Mild TBI OR (survival) 1.7 (95% CI 1.1-39) *p*0.01 Moderate TBI OR (survival) 1.4 (95% CI 1.2-3.1) *p*0.01 Severe TBI OR (survival) 1.2 (95% CI 0.8-2.9) *p*0.47 11(9-13) vs 9(8-10) *p*0.04 3(3-6) vs 4(3-5) *p*0.09	n	n	Relatively young population.

### Study characteristics

The eight studies identified for meta-analysis represented a heterogeneous cohort and total population of 105,675 patients; four pertained to traumatic brain injury (TBI) (*n* = 101,426) [13–16]⁠, three to general trauma (*n* = 4026) [17–19]⁠ and one to burn patients (*n* = 223) [20]⁠. Study size varied with the smallest including 188 patients and the largest over 100,000. Eight studies reported mortality data adequately for meta-analysis. No study verified medication adherence, minimum duration of therapy, or timing of last dose relative to injury, and only one study reported continuation of statin therapy following hospital admission [20]⁠.

Two identified the individual pre-morbid statins [12, 13]⁠, potentially allowing for corroboration of the 2010 Danish data [9]⁠. Three defined the a priori injury severity using a scale as part of the inclusion criteria [14, 17, 19]⁠. Only one TBI study specified isolated TBI [16]⁠. As such, exposure classification reflects documented statin prescription or reported use rather than confirmed pharmacologic exposure.

### Risk of bias and quality of evidence

**Fig. 2:**
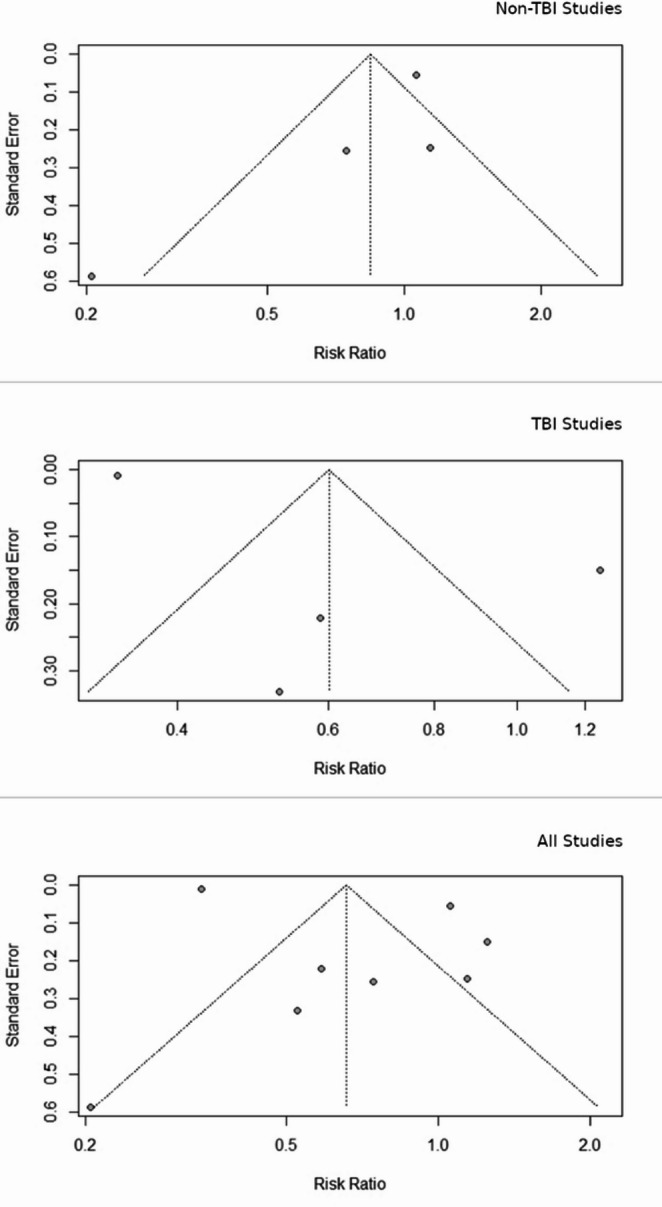
Funnel plots demonstrating risk of bias according to sub-group analysis

Significant and common flaws were lack of confidence in “assessment of exposure” and assessment of “presence or absence of prognostic factors”. As such all studies demonstrated a significant risk of bias (Fig. 2).

Using the NIH Quality Assessment Tool for Observational Cohort and Cross-Sectional Studies (10)⁠. Three [12, 13, 18]⁠ of the nine studies included in the systematic review were rated good quality. The remaining six [14–17, 20]⁠ rated fair.

All studies were retrospective and risk of bias was judged serious or very serious as studies relied on prescription-based exposure definitions without confirmation of adherence, duration, or continuation after admission. Thus, the overall GRADE assessment was “low.” No upgrading criteria were met.

### Primary outcome-mortality

**Fig. 3:**
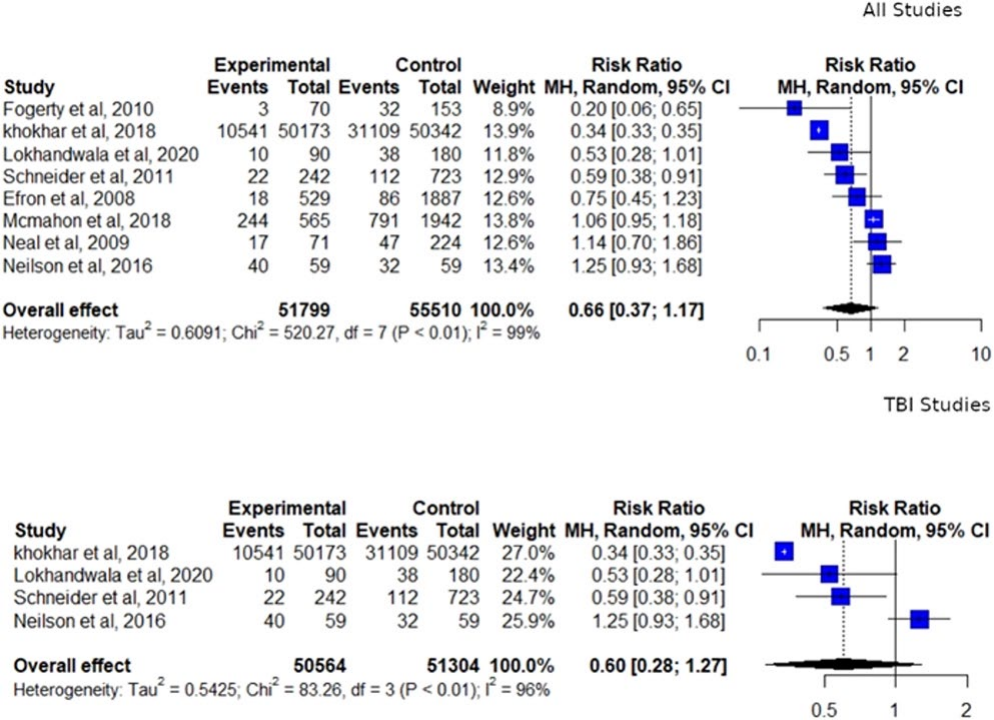
Forest plots of mortality risk according to sub-group analysis. (NB: I^2^ approaching 100% the pooled effect estimate should not be interpreted as evidence of a clinical effect, but rather as an exploratory summary of the direction and magnitude of association across heterogeneous observational cohorts.**)**

In the limited meta-analysis of the entire cohort (I2 = 98.7%) yielded an overall mortality risk ratio of 0.66 [95% CI 0.37–1.17, Fig. 3]. The TBI sub-group (I2 = 96.4%) had a risk ratio of 0.60 [95% CI 0.28–1.27]. As the 2016 Neilson et al. study [15]⁠ was the only study that lay clearly to the right of 1 and had significant limitations, a second analysis was performed treating it as an outlier, with the initial analysis intended as the primary analysis.

In this exploratory analysis, the cohort became significantly less heterogeneous (I2 = 74%) and produced a risk ratio of 0.44 [95% CI 0.29–0.68]. The non-TBI sub-group demonstrated significant heterogeneity (I2 = 70%) and a risk ratio of 0.84 [95% CI 0.56–1.27].

### Secondary outcomes

Although reporting of length-of-stay and complication rates was too inconsistent for secondary analysis, they warrant discussion.

### Infection and sepsis

Three studies (17–19)⁠ reported rates of infection or sepsis. Fogerty reported 50% decrease in rates of septic shock [*p* = 0.15], though the general rate of infection remained similar in statin-users compared to non-users [20]⁠. Neal et al. found a significant [*p* = 0.04] increase in the rates of multi-organ failure in patients with prior statin use [18]⁠, though nosocomial infection rates did not differ. Efron identified both infection and septic shock were higher in statin-users [*p* = 0.04] [19]⁠, and statin-users were more likely to present in shock.

### Lengths of stay

Three studies reported both ICU and hospital length-of-stay [16–18]⁠. Neither Lokhandwala nor Neal demonstrated a significant difference in either category [16, 18]⁠. Statin-users in the study conducted by McMahon and colleagues stayed longer in both the ICU and the hospital [17]⁠. In contrast to Neal et al. [18]⁠ who found no difference in the number of ventilator days, McMahon reported a significant increase in ventilator days in statin-users [17]⁠.

### Neurological outcomes

All four studies examining statin use in TBI [12–14, 16]⁠ reported data on neurological outcomes. Although not demonstrable at the three month mark, Schneider reported better neurological outcomes at 12 months, measured by the GOS-E (Glasgow Outcome Score Extended) [14]⁠. Lokhandwala and colleagues showed both significantly better outcomes in statin-users as measured by the GOS-E and less frequent admissions to skilled-nursing facilities [16]⁠. Neilson reported no reduction in poor outcomes when comparing statin-users and non-users [15]⁠. The only study pertaining to TBI that did not report GOS-E was the only one to examine the rate of CVI (cerebrovascular incident), ADRD (Alzheimer’s Disease Related Dementias) and Depression [12]⁠. Though all statins were associated with a lower risk of stroke (ischaemic or haemorrhagic), atorvastatin and simvastatin were associated with the largest risk reduction [12]. All except fluvastatin were linked to a lower incidence of ADRD and post-TBI depression.

One article not included in the review but deserves mention was the use of statins in spinal cord injury [21]. This retrospective study assessed recovery in motor function in patients using the International Standards for Neurological Classification of Spinal Cord Injury (ISNCSCI) motor subscore. Twenty-five out of eighty-three patients were on statins pre-morbidly and had a significant reduction in motor subscore at the two month mark. The study concluded there may be pleiotropisms of statins that may affect neurological and cognitive recovery but larger scale investigation is necessary.

### Venous thromboembolism

Patients in the study conducted by McMahon and colleagues were at increased risk of VTE (Venous Thromboembolism) if on statins prior to admission [5.8% vs. 3.3%, p = < 0.01] [17]⁠. This increased risk was maintained in cases of progression from VTE to DVT (Deep Vein Thrombosis) [4.2% vs. 2.1%, p = < 0.01]. Similarly they reported a higher incidence of IVC Filter (Inferior Vena Cava Filter) placement in statin recipients [2.5% vs. 1.2%] [17]⁠. No mention was made of VTE-prophylaxis or of the indication for the surprisingly high rate of IVCF insertion. Further rates of VTE and DVT in both groups seem to lie well within normal rates of occurrence in the setting of an established VTE-prophylaxis protocol [22–24]⁠.

## Discussion

In the context of heterogeneity of data, high risk of bias, and low certainty of results, this limited meta-analysis did not demonstrate a statistically significant benefit or even association to pre-morbid statin therapy in trauma. A recurring observation across the traumatic brain injury literature was a tendency toward more favourable neurological or survival outcomes among patients classified as pre-morbid statin users. However, this pattern was not uniform, nor was it consistently supported across other trauma phenotypes. In contrast, studies reporting infectious outcomes and lengths of stay yielded discordant findings, with reports of reduced, unchanged, and increased risks observed across different cohorts. Of the three studies reporting infection rate one [20]⁠ reported a decrease in the rate of infection, one [18]⁠ no difference, while another [19]⁠ reported an increase in infection rates. Similarly, two studies found no difference regarding lengths-of-stay [16, 18]⁠ in contrast to a single study (16)⁠ demonstrating increased lengths-of-stay in statin users.

This variability underscores the absence of a coherent signal across outcomes and populations, and highlights the difficulty of drawing inferences from pooled estimates derived from clinically disparate settings, including isolated TBI, polytrauma, and burns.

The only statistically significant finding was identified through exclusion of the Neilson et al. study [15]⁠ which shifted both the overall effect and the TBI subgroup to the left. However, this is problematic as it removes a possibly legitimate finding. By excluding the study, the high weighting it was allotted (25.9%) was redistributed primarily to Khokhar et al. [12], effectively doubling its weighting. Although Neilson et al. [15]⁠ found a non-significant survival trend in statin-naive patients following TBI, the study had limitations. It was small and examined a specific south-east Asian population. Neilson and colleagues [15] postulated that this population may respond differently to statins at lower doses than western patients. Additionally, pre-morbid statin users were more likely to have concomitant, severe extracranial injuries with a significantly higher rate of third ventricle effacement. It is improbable either of these can be attributed to pre-morbid statin therapy. The exclusion of this study while performed post analysis should be interpreted as an exploratory analysis to find associations, especially in the context of an already heterogeneous population.

This meta-analysis was subject to several limitations which substantially constrain causal interpretation and reinforce that the present findings should be regarded as associative and hypothesis-generating only. Key amongst them was the uncertainty in exposure definition. In most included studies, pre-morbid statin use was defined using prescription or administrative records, resulting in prescription-as-exposure misclassification, with no confirmation of medication adherence, minimum duration of therapy, or timing of last dose relative to injury. In addition, continuation or discontinuation of statin therapy after hospital admission was rarely reported, further obscuring true pharmacologic exposure. These limitations introduce several well-recognised sources of bias. Amongst them is immortal time bias, wherein classification as a statin user requires survival to exposure ascertainment and may preferentially assign early deaths to the non-statin group. Healthy-user bias could also be present, as patients prescribed long-term statins often differ systematically from non-users in baseline health status, healthcare utilisation, preventive care engagement, and socioeconomic factors. Collectively, these biases may generate spurious protective associations/confounding factors independent of any pharmacologic effect and are inherent to retrospective observational designs relying on prescription-based exposure definitions. Additional limitations include the high degree of statistical heterogeneity observed in this review suggests random variation rather than genuine differences. Included studies differed substantially with respect to trauma mechanism and severity, age distribution, comorbidity burden, health system context, and analytic methods. With heterogeneity approaching 100%, the pooled estimates are of limited value. These differences limit interpretability of any pooled effect while questioning any underlying treatment effect.

This isn’t to say statins have no effect. Mraiche and colleagues [25]⁠ were able to identify some direct effects of statins within 30 min of administration, at least one other study suggests that up to two weeks may be required for the complete onset of pleiotropic effects [26]⁠. Whilst unlikely that a significant portion of the study population had been under statin therapy for less than two weeks at the time of enrolment, no study reported a minimum duration of statin therapy. However, these associations cannot be assumed to reflect pleiotropic anti-inflammatory or endothelial effects. Rather, they may represent residual confounding related to baseline health and healthcare engagement that is incompletely captured by multivariable adjustment or propensity-based methods.

## Conclusion

This study demonstrated a non-statistically significant trend towards improved mortality with pre-morbid statin use following serious injury. It is possible that the small size of most of the included studies resulted in this meta-analysis being underpowered as any true beneficial effect is likely small.

As such, we feel the results of our study should not be used to support claims of clinical benefit or therapeutic efficacy, but rather to inform hypothesis generation and future study design.

## Supplementary Information

Below is the link to the electronic supplementary material.

Supplementary Material 1 (DOCX 102 KB)

## Data Availability

No datasets were generated or analysed during the current study.
