# Supplementary material for: Pre-morbid statin use and mortality in trauma: a systematic review and meta-analysis
Source: Langenbecks Arch Surg. 2026 Apr 2;411(1):138. doi: 10.1007/s00423-026-04011-8 (PMC13167902; doi:10.1007/s00423-026-04011-8)
Supplement: Supplementary file 1 — Supplementary Material 1 (DOCX 102 KB) [file 423_2026_4011_MOESM1_ESM.docx]

**Appendix S1**

**Pleiotropic Effects of Statins**

Although it is not fully understood how statins impact morbidity and mortality following injury, it may be via the pleiotropic effects. Statins achieve cholesterol lowering effects via reversible inhibition of HMG-CoA reductase, which catalyses the rate-limiting conversion of HMG-CoA to mevalonic acid [1–3]. The suppression of mevalonic acid synthesis then reduces downstream synthesis of the isoprenoids farnesyl pyrophosphate [FPP] and geranylgeranyl pyrophosphate (GGPP) [4]⁠. These isoprenoids are used in the synthesis of G-proteins, Ras, and Ras-like proteins, integral to signalling pathways. As they occur via various mechanisms, it is difficult to determine the onset time for pleiotropic effects. Although some effects manifest shortly after administration [5], it is likely that others require up to two weeks of statin therapy to manifest [6]⁠. A limited graphical overview of pleiotropic effects is found in Figure 1.

**Fig 1** The Pleiotropic Effect of Statins


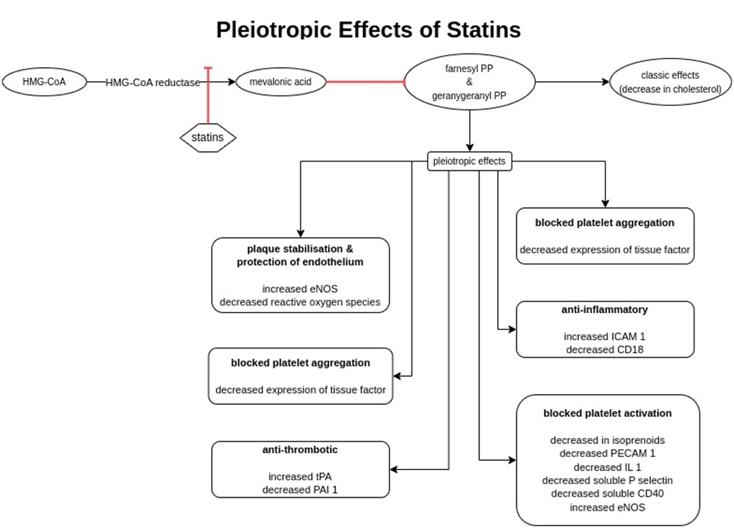


The following sections discuss the pleiotropic effects of statins as a possible mechanism affecting outcomes following trauma.

## Anti-inflammatory Effects of Statins

C-reactive protein (CRP) is a non-specific marker of inflammation frequently used to monitor therapy in the intensive care unit (ICU) [7,8)]. However, CRP is more than a marker of inflammation; it both stimulates the production of, and is stimulated by, interleukin-6 (lL6), amplifying the inflammatory cascade [9–11)]. Additionally, CRP promotes plasminogen activator-1 expression leading to complement activation, an increase in the expression of intracellular adhesion molecules and a decrease in endothelial nitric oxide synthase (eNOS) [12,13)]. This either initiates or amplifies the inflammatory cascade, promoting vascular dysfunction (discussed in the next section). Although the mechanism remains unclear, statins appear to significantly reduce CRP levels independent of their cholesterol-lowering effect [14)]. Other cytokines involved in inflammation important to statins are interleukin 8 (IL-8), tumour necrosis factor alpha (TNF-α), and nuclear factor kappa B (NF-κB), the levels of which correlate to the severity of systemic inflammatory response syndrome (SIRS) [15,16)].

Neutrophil adhesion, migration and survival after migration are dependent upon multiple cytokines and adhesion molecules, like intracellular adhesion molecule-1 (ICAM-1), protein kinase B pathway PI-3K, IL-6, IL-8, TNF-α, CD11b and CD18 [17)]. Statins are associated with a decrease in TNF-α, IL-6, IL-8 and CD11b in a cholesterol-independent fashion, possibly modulating neutrophil adhesion, migration and survival [15,18)]. NF-κB synthesis, promoted by TNF-α [19)], also induces these pro-inflammatory cytokines and upregulation of adhesion molecules as a result of oxidative stress and toll-like receptor-4 (TLR-4) signalling [20–22)]. Ajmieh and colleagues [23]⁠ showed atorvastatin inhibited TLR-4 expression, blocking the activation of NF-κB and its downstream effects. Statins seem also to inhibit neutrophil adhesion via suppression of lymphocyte function-associated antigen-1 (LFA-1), ICAM-1 and CD11b [18,24,25)]. Via a mechanism at least partially dependent upon the inhibition of the Rho-kinase pathway, statins also reduce neutrophil migration within two weeks of statin initiation [25,26)]. Many of these cytokines also contribute to the formation of proteolytic enzymes, microvascular thrombi and reactive oxygen species [27,28)], which are partly countered by the statin-induced eNOS upregulation [23]⁠. The eNOS upregulation additionally impairs apoptosis [29]⁠.

Within the trauma setting, an increase in inflammatory cytokines may already be demonstrable at the time of admission [30,31]. Continued elevated cytokine levels at 24hrs, 48hrs and 72hrs are associated with adverse outcomes, including adverse cardiac events [32]⁠.

Through various mechanisms, statins appear to reduce the activation and amplification of the inflammatory cascade via the suppression of pro-inflammatory cytokines. Subsequent neutrophil adhesion and migration is also inhibited. This modulation of innate inflammation may reduce secondary injury after trauma.

## Endovascular Effects of Statins

The primary mechanism by which statins affect the endovascular system appears to be eNOS upregulation [23,29]⁠. One way this occurs is through induction of eNOS via the protein kinase B pathways PKA and PI-3K/AKT [33)]. Activation of AKT may additionally confer increased endothelial cell survival and promote angiogenesis via vascular endothelial growth factor (VEGF) [33)]. Further, statins likely have the ability to directly stimulate cell surface scavenger receptor-B1, a g-protein coupled receptor leading to eNOS synthesis [34)] independently of HMG-CoA reductase inhibition. Regardless of the mechanism, the enhanced eNOS production leads to vasodilation and decreased platelet aggregation [35,36)], promoting vascular patency and improving perfusion. Statins may also promote increased endothelial production of tissue plasminogen activator (tPA) [37,38]⁠, perhaps minimising microvascular disruption. Statins may also actively inhibit vasoconstriction by suppressing the effects of the vasoconstrictor endothelin-1 and its synthesis by blocking transcription via the Rho-kinase pathway [5,25]⁠. Platelet activity is likely mediated via reduced synthesis of thromboxane-a2, a reduction in a2-adrenergic receptor density and a decrease in platelet cytosolic calcium [39–41]⁠. Statins appear to promote vascular patency and perfusion via vasodilation, inhibition of vasoconstriction and decreased platelet activation.

## Effects of Statins in Infection and Multi-organ Dysfunction

In one trial examining the use of statins as an adjunctive therapy in sepsis, serum IL-6 was found to be significantly lower at admission in patients previously undergoing statin treatment [42]⁠. A strong correlation was found between IL-6 levels and ICU, 28-day, 90-day mortality, and the sequential organ failure assessment (SOFA) score. Although serum IL-6 levels were similar in all patients during ICU treatment, those with pre-morbid statin therapy demonstrated improved 28-day mortality. De novo statin therapy initiated after admission was associated with a significant reduction of ICU length of stay. A second prospective observational study found a 16% risk reduction for severe sepsis in patients with pre-morbid statin therapy [43] and a reduction in relative risk of ICU admission.

A retrospective analysis of patients with multi-organ dysfunction syndrome (MODs) demonstrated a clear benefit of pre-morbid statin therapy in patients admitted to the ICU at 28 days [44)]. The mortality benefit remained significant at hospital discharge.

Although unproven, the mechanisms by which statins potentially improve outcomes in sepsis and MODs are likely related to those outlined above.

# Supplemental References

1. Liao JK, Laufs U. Pleiotropic Effects of Statins. Annu Rev Pharmacol Toxicol. 2005 Sep;45(1):89–118.

2. Rodwell VW. Regulation of HMG-CoA Reductase. :74.

3. Istvan ES. Structural Mechanism for Statin Inhibition of HMG-CoA Reductase. Science (80- ). 2001;292(5519):1160–4.

4. Goldstein JL, Brown MS. Regulation of the mevalonate pathway. Nature. 1990;343(6257):425–30.

5. Mraiche F, Cena J, Das D, Vollrath B. Effects of statins on vascular function of endothelin-1: {Statins} inhibit vascular actions of endothelin-1. Br J Pharmacol. 2005;144(5):715–26.

6. Lazar HL, Bao Y, Zhang Y, Bernard SA. Pretreatment with statins enhances myocardial protection during coronary revascularization. J Thorac Cardiovasc Surg. 2003;125(5):1037–42.

7. Ranzani OT, Prada LF, Zampieri FG, Battaini LC, Pinaffi J V, Setogute YC, et al. Failure to reduce C-reactive protein levels more than 25% in the last 24 hours before intensive care unit discharge predicts higher in-hospital mortality: A cohort study. J Crit Care. 2012;27(5):525.e9--525.e15.

8. Yentis SM, Soni N, Sheldon J. C-reactive protein as an indicator of resolution of sepsis in the intensive care unit. Intensive Care Med. 1995;21(7):602–5.

9. Jones SA, Novick D, Horiuchi S, Yamamoto N, Szalai AJ, Fuller GM. C-reactive Protein: A {Physiological} Activator of Interleukin 6 Receptor Shedding. J Exp Med. 1999;189(3):599–604.

10. Arnaud C, Burger F, Steffens S, Veillard NR, Nguyen TH, Trono D, et al. Statins Reduce Interleukin-6-Induced C-Reactive Protein in Human Hepatocytes: New Evidence for Direct Antiinflammatory Effects of Statins. Arterioscler Thromb Vasc Biol. 2005;25(6):1231–6.

11. Baumann H, Gauldie J. The acute phase response. Immunol Today. 1994;15(2):74–80.

12. Bhakdi S, Torzewski M, Klouche M, Hemmes M. Complement and Atherogenesis: Binding of CRP to Degraded, Nonoxidized LDL Enhances Complement Activation. Arterioscler Thromb Vasc Biol. 1999;19(10):2348–54.

13. Torzewski J. Processes in atherogenesis: complement activation. Atherosclerosis. 1997;132(2):131–8.

14. Albert MA, Danielson E, Rifai N, Ridker PM. Effect of Statin Therapy on C-Reactive Protein Levels: the pravastatin inflammation/CRP evaluation (PRINCE): a randomized trial and cohort study**.** JAMA. 2001 Jul 4;286(1):64-70

15. Chello M, Patti G, Candura D, Mastrobuoni S, Di Sciascio G, Agrò F, et al. Effects of atorvastatin on systemic inflammatory response after coronary bypass surgery: Crit Care Med. 2006;34(3):660–7.

16. Jaffer U, Wade RG, Gourlay T. inflammatory response syndrome: a review. 2010;2:15.

17. McGettrick HM. Chemokine- and adhesion-dependent survival of neutrophils after transmigration through cytokine-stimulated endothelium. J Leukoc Biol. 2006;79(4):779–88.

18. Weber C, Erl W, Weber KSC, Weber PC. HMG-CoA Reductase Inhibitors Decrease CD11b Expression and CD11b-Dependent Adhesion of Monocytes to Endothelium and Reduce Increased Adhesiveness of Monocytes Isolated From Patients With Hypercholesterolemia. J Am Coll Cardiol. 1997 Nov;30(5):1212–7.

19. Albensi BC. What Is Nuclear Factor Kappa B (NF-κB) Doing in and to the Mitochondrion? Front Cell Dev Biol. 2019 Aug;7:154.

20. Jaeschke H, Lemasters JJ. Apoptosis versus oncotic necrosis in hepatic ischemia/reperfusion injury. Gastroenterology. 2003;125(4):1246–57.

21. Temkin V, Karin M. From death receptor to reactive oxygen species and c-Jun N-terminal protein kinase: the receptor-interacting protein 1 odyssey. Immunol Rev. 2007;220(1):8–21.

22. Teoh NC, Farrell GC. Hepatic ischemia reperfusion injury: Pathogenic mechanisms and basis for hepatoprotection. J Gastroenterol Hepatol. 2003 Aug;18(8):891–902.

23. Ajamieh H, Farrell G, Wong HJ, Yu J, Chu E, Chen J, et al. Atorvastatin protects obese mice against hepatic ischemia-reperfusion injury by Toll-like receptor-4 suppression and endothelial nitric oxide synthase activation: Atorvastatin and ischemia-reperfusion injury. J Gastroenterol Hepatol. 2012 Aug;27(8):1353–61.

24. Chello M. Simvastatin attenuates leucocyte-endothelial interactions after coronary revascularisation with cardiopulmonary bypass. Heart. 2003;89(5):538–43.

25. Maher BM, Dhonnchu TN, Burke JP, Soo A, Wood AE, Watson RWG. Statins alter neutrophil migration by modulating cellular {Rho} activity--a potential mechanism for statins-mediated pleotropic effects? J Leukoc Biol. 2008;85(1):186–93.

26. Kinsella A, Raza A, Kennedy S, Fan Y, Wood AE, Watson RW. The impact of high-dose statin therapy on transendothelial neutrophil migration and serum cholesterol levels in healthy male volunteers. Eur J Clin Pharmacol. 2011 Nov;67(11):1103–8.

27. Kawahito K, Kobayashi E, Ohmori M, Harada K, Kitoh Y, Fujimura A, et al. Enhanced Responsiveness of Circulatory Neutrophils After Cardiopulmonary Bypass: Increased Aggregability and Superoxide Producing Capacity. Artif Organs. 2000;24(1):37–42.

28. Partrick DA, Moore EE, Fullerton DA, Barnett CC, Meldrum DR, Silliman CC. Cardiopulmonary Bypass Renders Patients at Risk for Multiple Organ Failure via Early Neutrophil Priming and Late Neutrophil Disability. J Surg Res. 1999 Sep;86(1):42–9.

29. Laufs U, La Fata V, Plutzky J, Liao JK. Upregulation of Endothelial Nitric Oxide Synthase by HMG CoA Reductase Inhibitors. Circulation. 1998;97(12):1129–35.

30. Torres LN, Sondeen JL, Ji L, Dubick MA, Torres Filho I. Evaluation of resuscitation fluids on endothelial glycocalyx, venular blood flow, and coagulation function after hemorrhagic shock in rats. J Trauma Acute Care Surg. 2013 Nov;75(5):759–66.

31. De’Ath HD, Rourke C, Davenport R, Manson J, Renfrew I, Uppal R, et al. Clinical and biomarker profile of trauma-induced secondary cardiac injury. Br J Surg. 2012 Apr;99(6):789–97.

32. De’Ath HD, Manson J, Davenport R, Glasgow S, Renfrew I, Davies LC, et al. Trauma-Induced Secondary Cardiac Injury Is Associated With Hyperacute Elevations in Inflammatory Cytokines. Shock. 2013;39(5):415–20.

33. Kureishi Y, Luo Z, Shiojima I, Bialik A, Fulton D, Lefer DJ, et al. The HMG-CoA reductase inhibitor simvastatin activates the protein kinase Akt and promotes angiogenesis in normocholesterolemic animals. Nat Med. 2000 Sep;6(9):1004–10.

34. Datar R, Kaesemeyer W, Chandra S, Fulton D, Caldwell R. Acute activation of eNOS by statins involves scavenger receptor-B1, G protein subunit Gi, phospholipase C and calcium influx: Acute activation of eNOS by statins. Br J Pharmacol. 2010 Aug;160(7):1765–72.

35. Ignarro LJ, Buga GM, Wood KS, Byrns RE, Chaudhuri G. Endothelium-derived relaxing factor produced and released from artery and vein is nitric oxide. Proc Natl Acad Sci. 1987;84(24):9265–9.

36. Radomski MW, Rees DD, Dutra A, Moncada S. S-nitroso-glutathione inhibits platelet activation in vitro and in vivo. Br J Pharmacol. 1992 Nov;107(3):745–9.

37. Essig M, Nguyen G, Prié D, Escoubet B, Sraer JD, Friedlander G. 3-Hydroxy-3 Methylglutaryl Coenzyme A Reductase Inhibitors Increase Fibrinolytic Activity in Rat Aortic Endothelial Cells: Role of Geranylgeranylation and Rho Proteins. Circ Res. 1998;83(7):683–90.

38. Asahi M, Huang Z, Thomas S, Yoshimura S ichi, Sumii T, Mori T, et al. Protective Effects of Statins Involving Both eNOS and tPA in Focal Cerebral Ischemia. J Cereb Blood Flow \& Metab. 2005;25(6):722–9.

39. Notarbartolo A, Davı̀ G, Averna M, Barbagallo CM, Ganci A, Giammarresi C, et al. Inhibition of Thromboxane Biosynthesis and Platelet Function by Simvastatin in Type IIa Hypercholesterolemia. Arterioscler Thromb Vasc Biol. 1995;15(2):247–51.

40. Sang KHLQ, Levenson J, Megnien JL, Simon A, Devynck MA. Platelet Cytosolic Ca and Membrane Dynamics in Patients With Primary Hypercholesterolemia: Effects of Pravastatin. Arterioscler Thromb Vasc Biol. 1995;15(6):759–64.

41. Baldassarre D, Mores N, Colli S, Pazzucconi F, Sirtori CR, Tremoli E. Platelet α2-adrenergic receptors in hypercholesterolemia: Relationship between binding studies and epinephrine-induced platelet aggregation. Clin Pharmacol & Ther. 1997;61(6):684–91.

42. Kruger P, Bailey M, Bellomo R, Cooper DJ, Harward M, Higgins A, et al. A Multicenter Randomized Trial of Atorvastatin Therapy in Intensive Care Patients with Severe Sepsis. Am J Respir Crit Care Med. 2013 Apr;187(7):743–50. 43. Almog Y, Shefer A, Novack V, Maimon N, Barski L, Eizinger M, et al. Prior Statin Therapy Is Associated With a Decreased Rate of Severe Sepsis. Circulation. 2004 Aug;110(7):880–5.

44. Schmidt H, Hennen R, Keller A, Russ M, Müller-Werdan U, Werdan K, et al. Association of statin therapy and increased survival in patients with multiple organ dysfunction syndrome. Intensive Care Med. 2006 Aug;32(8):1248–51.
